# Virtual Screening of Hepatitis B Virus Pre-Genomic RNA as a Novel Therapeutic Target

**DOI:** 10.3390/molecules28041803

**Published:** 2023-02-14

**Authors:** Lukasz T. Olenginski, Wojciech K. Kasprzak, Solomon K. Attionu, Bruce A. Shapiro, Theodore K. Dayie

**Affiliations:** 1 Department of Chemistry and Biochemistry, University of Maryland, College Park, MD 20742, USA; 2 Bioinformatics and Computational Science Program, Frederick National Laboratory for Cancer Research, National Cancer Institute, Frederick, MD 21702, USA; 3 RNA Biology Laboratory, National Cancer Institute, Frederick, MD 21702, USA

**Keywords:** virtual screening, drug discovery, molecular dynamics, RNA, hepatitis B virus

## Abstract

The global burden imposed by hepatitis B virus (HBV) infection necessitates the discovery and design of novel antiviral drugs to complement existing treatments. One attractive and underexploited therapeutic target is ε, an ~85-nucleotide (nt) *cis*-acting regulatory stem-loop RNA located at the 3′- and 5′-ends of the pre-genomic RNA (pgRNA). Binding of the 5′-end ε to the viral polymerase protein (P) triggers two early events in HBV replication: pgRNA and P packaging and reverse transcription. Our recent solution nuclear magnetic resonance spectroscopy structure of ε permits structure-informed drug discovery efforts that are currently lacking for P. Here, we employ a virtual screen against ε using a Food and Drug Administration (FDA)-approved compound library, followed by in vitro binding assays. This approach revealed that the anti-hepatitis C virus drug Daclatasvir is a selective ε-targeting ligand. Additional molecular dynamics simulations demonstrated that Daclatasvir targets ε at its flexible 6-nt priming loop (PL) bulge and modulates its dynamics. Given the functional importance of the PL, our work supports the notion that targeting ε dynamics may be an effective anti-HBV therapeutic strategy.

## 1. Introduction

Globally, hepatitis B virus (HBV) chronically infects over 300 million people, leading to ~555,000 deaths per year [1]. In fact, HBV accounts for ~23 and ~40% of all cases of cirrhosis (i.e., sever liver damage) and hepatocellular carcinoma (HCC), respectively [1,2]. The global burden imposed by HBV therefore necessitates the discovery and design of novel antiviral drugs. The Food and Drug Administration (FDA)-approved treatments of chronic HBV infection are interferon (IFN)-α and nucleos(t)ide reverse transcriptase inhibitors (NRTIs). Unfortunately, these treatments are not curative and involve lifelong therapy and/or adverse effects [3,4,5,6,7], requiring the need for alternative anti-HBV treatments. HBV is a member of the hepadnaviral family and is the smallest animal-infecting DNA virus, with a genome of only 3.2 kilobases [8,9,10,11,12]. The HBV genome is partially double-stranded, relaxed circular DNA and is covalently attached to a multifunctional viral polymerase protein (P) comprising four domains [13,14,15,16,17,18,19]. Viral replication is initiated by the binding of P to the 5′-copy of epsilon (ε), an ~85-nucleotide (nt) *cis*-acting regulatory stem-loop RNA located at the 3′- and 5′-ends of the pre-genomic RNA (pgRNA) [20,21,22,23,24]. This interaction triggers packaging of the pgRNA and P into subviral core particles [13,25] and the initiation of reverse transcription [26,27,28,29], making the ε–P complex an attractive therapeutic target for the early intervention of HBV replication. 

**Figure 1:**
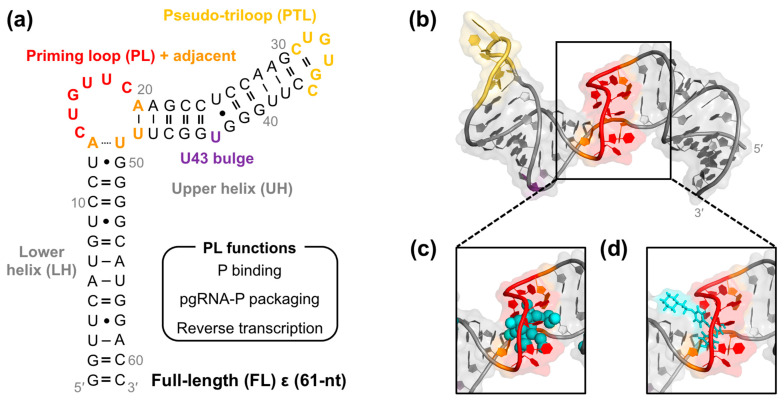
HBV ε as an attractive therapeutic target. (**a**) Secondary structure of a 61-nt ε containing the entire stem-loop region, designated full-length (FL), with structural regions abbreviated and color-coded. (**b**) Solution NMR structure of FL ε (PDB 6var) [30]. (**c**) The most probable FL ε ligand cavity, as determined from RNACavityMiner [31], is shown in cyan spheres. (**d**) The top-ranked docking pose of Raloxifene to FL ε is shown in cyan sticks, as previously described [30]. Given our previous computational docking [30], all structure representations in (**b**–**d**) are FL ε NMR conformer 3 (ε R3) and are colored as in (**a**).

However, the lack of structural data on P prevents the structure-informed design of anti-HBV therapeutics. Our recent solution nuclear magnetic resonance (NMR) spectroscopy structure of a 61-nt ε [30] (Figure 1a,b) presents a necessary step in this direction. This ε construct contains the entire stem-loop region (Figure 1a) and will therefore be referred to as full-length (FL) ε throughout the text. Initial structural analysis of FL ε indicated that its 6-nt priming loop (PL) bulge forms a binding pocket that may be amenable to small molecule targeting (Figure 1b). Computational predictions [31] further support this notion, suggesting that the most probable ligand cavity within ε is its PL (Figure 1c). In agreement with this hypothesis, we identified Raloxifene and other selective estrogen receptor modulators (SERMs) as the first class of ε-targeting ligands [30] that selectively bind FL ε at its flexible [30,32] PL (Figure 1d). Given that the PL is required for P binding [33,34], pgRNA–P packaging [22,23,27,29,33,34], and reverse transcription [27,29,33] (Figure 1a), ligands that target this motif may have an inhibitory effect. Unfortunately, the SERMs were unable to inhibit the early stages of HBV reverse transcription [35], which likely reflects their low affinity (30–110 µM) as compared to the significantly higher affinity (low nM) of P for ε. Taken together, these studies motivate the need for additional RNA-targeted drug discovery efforts.

As a first step toward identifying novel ε-targeting ligands, we carried out a structure-informed virtual screening (VS) against FL ε using an FDA-approved compound library, followed by in vitro binding assays. This approach revealed that the anti-hepatitis C virus (HCV) drug Daclatasvir is a selective ε-targeting ligand. To model the FL ε–Daclatasvir interaction, we employed computational docking and molecular dynamics (MD) simulations. Taken together, our data demonstrate that Daclatasvir selectively targets FL ε at its flexible [30,32] PL and modulates its dynamics. Given the functional importance of the PL [22,23,27,29,33,34] (Figure 1a), our work supports the notion that targeting ε dynamics may be an effective anti-HBV therapeutic strategy.

## 2. Results

### 2.1. Virtual Screen Strategy

To identify additional ligands that target the ε PL, we employed a structure-informed VS approach. Computational docking can provide complementary data and corroborating evidence to experimental binding assays. Moreover, VS dramatically reduces the time to generate lead compounds. However, VS is not without limitations, especially when targeting RNA. For example, docking to RNA targets is complicated for flexible RNAs such as FL ε [30,32] and ligand-induced conformational changes. One approach to overcome this challenge is to treat the RNA target as a conformational ensemble that is then subject to VS [36,37,38]. These ensembles can either be computationally derived or experimentally selected [39,40,41,42,43,44,45]. The initial success of the latter approach using an NMR-derived ensemble in a VS suggests a promising path forward for RNA [39,40]. However, the utility of this method is predicated on having robust experimental NMR restraints, such as residual dipolar couplings (RDCs) and the Nuclear Overhauser effect (NOE). Unfortunately, these data are sparse for FL ε [30] due to its large size. To partially address the inherent dynamics [30,32] of FL ε, we instead used a rigid dock VS, followed by MD simulations.

### 2.2. Lead Compound Generation

The first step in our VS was receptor preparation and compound library selection. Based on our previous computational docking [30], we used FL ε R3 (PDB 6var) [30] as the receptor. We then selected an FDA-approved library curated in the ZINC15 database [46,47,48] to avoid additional lead compound selection steps such as ADMET [49,50,51] and Lipinski’s rule of five [52,53]. The former refers to absorption, distribution, metabolism, excretion, and toxicity and is highly predictive of drug efficacy and safety [49,50,51], whereas Lipinski’s suggestions state that successful drug candidates typically violated no more than one of the following considerations: ≤five hydrogen bond donors, ≤10 hydrogen bond acceptors, molecular weight ≤ 500 Daltons, and a logP ≤ 5.0 [52,53]. Our assumption is that FDA-approved drugs already have good drug-like properties, which paradoxically is not always true. Nevertheless, the value of our VS is that it can be easily repeated with a different compound library to identify new lead compounds, if needed.

**Figure 2:**
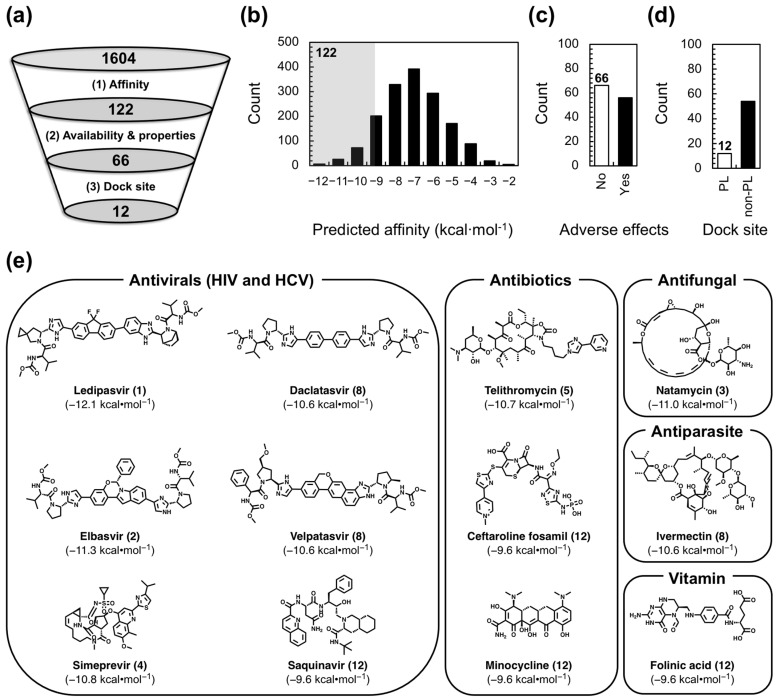
Virtual screen (VS) lead compound selection. (**a**) Schematic of our lead compound selection criteria, starting from the 1604 compound FDA-approved library. (**b**) Histogram showing all VS compounds sorted by their predicted affinities. The shaded region contains the 122 compounds with a predicted affinity higher than Raloxifene. (**c**) Plot of adverse effects (i.e., yes or no) for the 122 compounds that met the first selection criteria. (**d**) Plot of compound dock site (i.e., PL or non-PL) for the 66 compounds that met the first two selection criteria. For plots in (**c**,**d**), data shown in white represent compounds that met the given selection criteria. (**e**) Structure of VS-identified lead compounds separated by use, with predicted affinities to FL ε R3 and rank shown in parentheses.

With our receptor and compound library in place, we carried out our VS to identify FDA-approved drugs that selectively target FL ε and may therefore be repurposed as anti-HBV therapeutics. We opted to use AutoDock Vina [54] in the PyRx open-source software package [55] over more sophisticated RNA–ligand docking programs [56] because we prioritized the rapid identification of lead compounds over accurate binding pose predictions, which are less important given that we will experimentally verify the results of our VS. We employed selection criteria based on affinity, commercial availability and drug-like properties, and dock site (Figure 2a) to identify the lead compounds from our 1604-compound library. As our first selection step, we used the predicted affinity (−9.5 kcal·mol^−1^) of the already known [30] ε-targeting ligand Raloxifene as a cutoff to select the 122 compounds with a higher predicted affinity (Figure 2b). Raloxifene was chosen because we know it has an ε-binding affinity of ~70 µM [30] and assume that compounds with higher predicted affinities may also have higher experimental binding affinities. As such, this selection step increases the odds of finding lead compounds with low µM-to-high nM affinity. Next, we excluded all compounds that were not commercially available and/or had potential adverse effects (e.g., anticancer drugs; see Section 4) to proceed with the 66 compounds that would presumably be safe HBV treatments (Figure 2c). Finally, given the functional importance of the PL [22,23,27,29,33,34] (Figure 1a) and our previous computational [30,31] (Figure 1c) and experimental [30] (Figure 1d) data, in our final selection step, we chose the 12 compounds (Figure 2d) that reproducibly docked to the ε PL after repeated docking runs (Figure S1; see Section 4). 

Interestingly, our 12 VS-identified lead compounds show diversity in their structure and use (Figure 2e). For example, Ledipasvir, Elbasvir, Simeprevir, Daclatasvir, Velpatasvir, and Saquinavir are antivirals, all of which are anti-HCV drugs, except for Saquinavir, which targets human immunodeficiency virus (HIV) (Figure 2e). Telithromycin, Ceftaroline fosamil, and Minocycline, on the other hand, are antibiotics (Figure 2e). Lastly, Folinic acid, Natamycin, and Ivermectin are vitamin, antifungal, and antiparasitic compounds, respectively (Figure 2e). Our 12 VS-identified lead compounds also display predicted affinities to FL ε R3 ranging from −9.6 to −12.1 kcal·mol^−1^ (Figure 2e). Importantly, these newly discovered potential FL ε-targeting ligands can now be experimentally tested with in vitro binding assays to verify and quantify their interaction with FL ε. 

### 2.3. Daclatasvir Selectively Targets the ε Priming Loop

Our 12 VS-identified lead compounds (Figure 2e) were then screened for binding to FL ε with an in vitro dye-displacement binding assay (Figure S2). Here, FL ε was incubated with the fluorescent intercalator SYBR Green II dye, and the lead compounds were added at 500 µM. If our compounds bind FL ε, SYBR Green II fluorescence will decrease due to displacement of the dye. Surprisingly, none of the compounds caused fluorescence attenuation. Instead, some ligands (e.g., the antivirals; Figure 2e) actually led to an increase in fluorescence (Figure S3), indicating that these compounds either enhance the interaction between FL ε and the dye or bind the dye themselves. To rule out the former scenario, we repeated our dye-displacement assay with and without FL ε. Then, the non-RNA fluorescence signals were subtracted from the conditions with RNA to establish fluorescence decreases that are attributable to compounds that bind to FL ε (Figure S3). 

**Figure 3:**
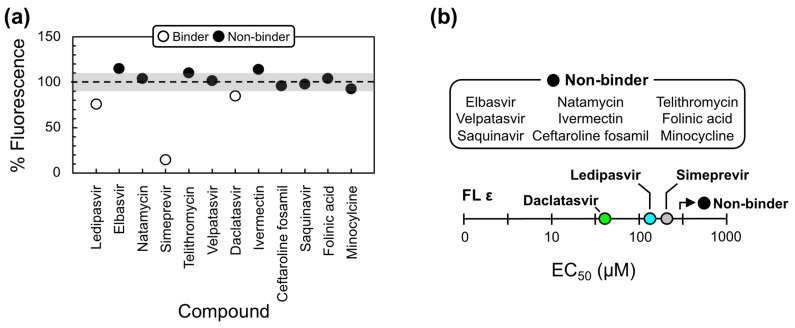
Lead compound binding to full-length (FL) ε. (**a**) Dye-displacement data for experimentally validating our 12 VS-identified lead compounds. % Fluorescence ≥100% (shown by the dashed line) indicates that the compound does not bind FL ε, whereas % Fluorescence <100% indicates that the compound does bind FL ε and displaces SYBR Green II. To avoid false positives, binding compounds were selected if they led to >10% fluorescence attenuation (shown by the lower shaded region). (**b**) Plot of the dye-displacement-derived EC_50_ values of our VS-identified lead compounds for FL ε. Full binding curves can be found in Figure S4. Non-binders show no evidence of fluorescent attenuation at the ligand concentrations used (i.e., EC_50_ > 500 µM).

Using this modified approach, nine compounds still showed no evidence of fluorescent attenuation, whereas three of the anti-HCV compounds (i.e., Ledipasvir, Simeprevir, and Daclatasvir) did (Figure 3a). These data suggest that the previous fluorescence increase was a result of SYBR Green II binding to the antiviral compounds, which is likely facilitated by the potential to form π-stacking interactions with their largely aromatic scaffolds (Figure 2e and Figure S2b). To assess the quality of our assay and provide a quantitative measure of binding, experiments were repeated by titrating increasing concentrations of each compound against FL ε. Since our compounds have to compete for RNA-binding with SYBR Green II, we measured the half-maximal effective concentration (EC_50_) values, which depend on the concentration and affinity of the dye. This analysis revealed that Simeprevir, Ledipasvir, and Daclatasvir bind FL ε with approximate EC_50_ values of 298, 145, and 62 µM, respectively (Figure 3b and Figure S4). 

**Figure 4:**
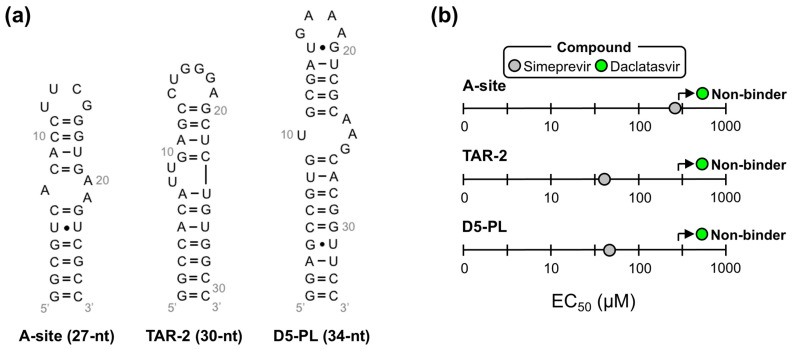
Daclatasvir selectively binds full-length (FL) ε. (**a**) Secondary structure of additional RNAs used to test the selectivity of Simeprevir and Daclatasvir binding to FL ε. (**b**) Plot of the dye-displacement-derived EC_50_ values of Ledipasvir, Simeprevir, and Daclatasvir for the RNAs shown in (**a**) Full binding curves can be found in Figure S5.

We then used our modified dye-displacement assay to test whether these three compounds bind additional RNA targets or are selective ε-ligands. To this end, additional RNAs with structural elements similar to ε (i.e., apical loops and internal bulges) were used: a 27-nt RNA from the decoding center of *Escherichia coli* ribosomal RNA (A-site), a 30-nt RNA from the transactive response element from HIV (TAR-2), and a 34-nt RNA from the self-splicing group II intron catalytic effector domain 5 from *Pylaiella littoralis* (D5-PL) (Figure 4a). Given that Ledipasvir was extremely insoluble, which would preclude the NMR experiments we intended to implement next, we only proceeded with Simeprevir and Daclatasvir. This analysis revealed that Simeprevir binds A-site, TAR-2, and D5-PL with approximate EC_50_ values of 436, 58, and 60 µM, respectively (Figure 4b and Figure S5). Since Simeprevir binds additional RNAs, some with lower EC_50_ values than FL ε (e.g., TAR-2 and D5-PL), it was no longer considered as a lead compound. Daclatasvir, on the other hand, showed no binding to the additional RNAs at the concentrations used (Figure 4b and Figure S5), demonstrating that it is a selective ε-targeting ligand.

**Figure 5:**
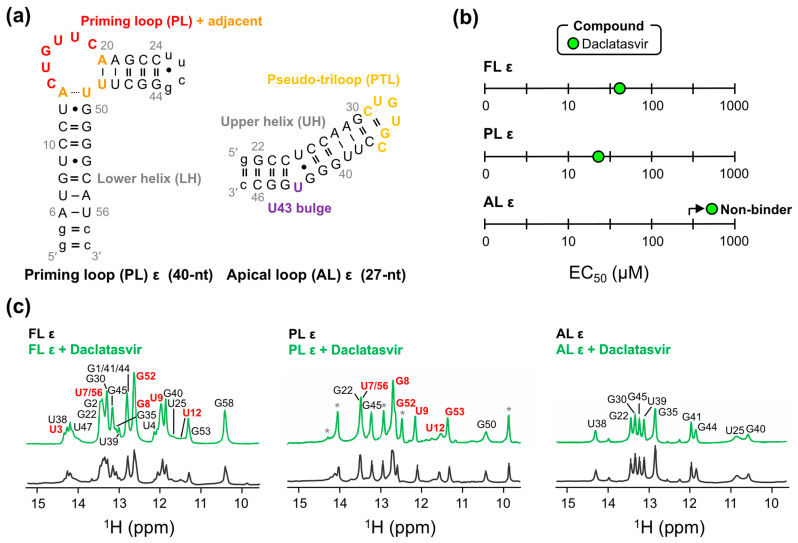
Mapping Daclatasvir binding to full-length (FL) ε. (**a**) Secondary structure of ε modular constructs with structural regions abbreviated and colored as in Figure 1a and using FL numbering. (**b**) Plot of the dye-displacement-derived EC_50_ values of Daclatasvir for PL ε and AL ε. Full binding curves can be found in Figure S6. (**c**) Imino ^1^H NMR spectra of all ε constructs titrated with Daclatasvir. NMR measurements were collected at 600 MHz and 25 °C. Imino proton resonance assignments are displayed on each spectrum, with non-native resonances (i.e., those not in FL ε) and resonances with CSPs shown as asterisks and in red, respectively. Due to resonance line broadening and overlap in FL ε, CSPs are more evident in PL ε titrations, though most CSPs are very small.

As a preliminary means of mapping the binding site of Daclatasvir to FL ε, we employed our modified dye-displacement assay a final time using two ε modular constructs (Figure 5a). The PL ε contains PL nucleotides C14–C19, four flanking base pairs on either side, an additional three base pairs to stabilize the lower helix (LH) and improve transcription, and a UUCG tetraloop to close the upper helix (UH) (Figure 5a). The apical loop (AL) ε comprises nucleotides G22–C46 of the UH and pseudo-triloop (PTL) with an additional terminal G:C base pair to improve transcription (Figure 5a). These experiments can therefore map Daclatasvir binding to distinct ε regions (i.e., LH, PL, PTL, and UH). Binding experiments with ε modular constructs demonstrated that Daclatasvir binds to PL ε but not AL ε (Figure 5b and Figure S6), suggesting that Daclatasvir binding is localized to the regions shared by the FL ε and PL ε constructs: the LH and PL. 

To verify our dye-displacement data, we employed NMR measurements. Due to Daclatasvir’s low solubility, we were limited to low concentration RNA samples and one-dimensional NMR experiments. We titrated Daclatasvir against all ε constructs (Figure 1a and Figure 5a) and monitored the chemical shift perturbations (CSPs) of imino protons (i.e., guanosine-H1 and uridine-H3) with ^1^H NMR. This analysis demonstrated that Daclatasvir titration only led to CSPs (and an increase in resonance intensities) in FL ε and PL ε (Figure 5c), suggestive of binding and in agreement with our dye-displacement data (Figure 5b). While these experiments cannot directly monitor changes in non-hydrogen-bonded (e.g., non-helical) regions of RNA due to the rapid exchange of imino protons with the solvent, all CSPs localize to nucleotides near the PL (i.e., upper part of the LH) (Figure 5c). These data are therefore consistent with Daclatasvir targeting ε at its PL (Figure S7). 

### 2.4. Modeling the Full-Length ε-Daclatasvir Complex

**Figure 6:**
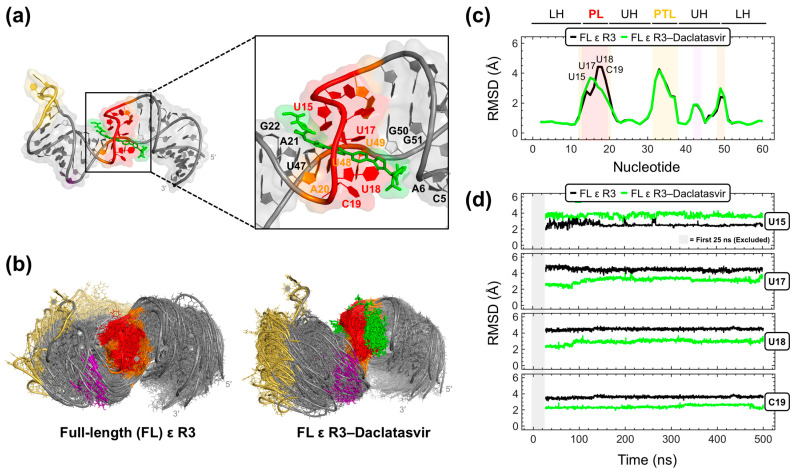
Computational modeling of the full-length (FL) ε R3–Daclatasvir complex. (**a**) Top-scored RNAPosers [58]-predicted Daclatasvir docking pose to FL ε R3 (PDB 6var) [30], with Daclatasvir shown in green sticks and interacting nucleotides labeled. (**b**) Structural overlay of PDB snapshots taken every 10 ns of the 500 ns FL ε R3 (left) and FL ε R3–Daclatasvir (right) MD trajectories. Structural ensembles in (**b**) are shown with backbone phosphorus atom alignments using PL nucleotides (i.e., A13–A20, U48, and U49). (**c**) All atom RMSD averaged over the 500 ns trajectories for FL ε R3 and the FL ε R3–Daclatasvir complex (see Section 4). (**d**) All atom RMSD for select FL ε nucleotides (i.e., U15 and U17–C19) calculated over the course of the MD runs (excluding the first 25 ns of equilibration, as shown by the gray shaded box). ε structural regions are abbreviated and colored as in Figure 1a.

Given that Daclatasvir was intractable to two-dimensional NMR experiments, we carried out computational docking and MD simulations as an alternative approach to model the FL ε–Daclatasvir interaction. To start, we ran repeated MD trajectories on the top-ranked Daclatasvir pose derived from AutoDock Vina. Three out of four simulations yielded quick ligand dissociations (Figure S8), suggesting an inaccurate docking pose and motivating the use of better-performing and more robust computational tools [56]. To this end, we employed rDock [57] to model Daclatasvir binding to FL ε R3. The docking poses generated by rDock were then resorted with RNAPosers [58] to find the most native pose. This analysis revealed that Daclatasvir selectively targets the ε PL with its core wedged between nucleotides U15 and U17-C19 and also contacting the adjacent A20-G22 and U47-G51 and C5 and A6 on the first turn of the LH (Figure 6a). Moreover, all 10 predicted poses dock to the ε PL with strong agreement (Figure S9), suggestive of an accurate prediction. It is important to note that the docking search space was not restricted to the ε PL, and therefore, our finding that the top-ranked Daclatasvir docking pose localizes to the PL, which is consistent with dye-displacement (Figure 5b) and NMR titration (Figure 5c) data (Figure S7), was not biased by the input parameters. 

As a way to partially address the inherent dynamics [30,32] of FL ε, we carried out 500 ns MD simulations on FL ε R3 and the FL ε R3–Daclatasvir complex with the top-scored pose derived from RNAPosers. In the latter trajectory, Daclatasvir remained stably bound to the RNA target at its PL and the upper part of the LH (Figure 6b), further suggesting a valid docking pose prediction (Figure 6a), and in agreement with our interpretation of the dye-displacement (Figure 5b) and NMR titration (Figure 5c) data (Figure S7). Representations of the MD-sampled ensembles of the unliganded and Daclatasvir-bound RNA demonstrate subtle differences in their dynamics (Figure 6b). This observation is more obvious when analyzing the root mean square deviation (RMSD) of all FL ε R3 nucleotide fluctuations throughout the MD runs compared to the starting NMR reference model (see Section 4). This analysis demonstrated that Daclatasvir modulates the flexibility of PL nucleotides (Figure 6c). Specifically, Daclatasvir increases the conformational variety (i.e., higher RMSD) of nucleotide U15 but lessens (i.e., lower RMSD) the motions of nucleotides U17-C19 (Figure 6c,d). Moreover, these Daclatasvir-induced dynamic modulations were retained for the duration of the MD trajectory (Figure 6d). Taken together, our combined experimental and computational approach identified the anti-HCV drug Daclatasvir as a selective ε-targeting ligand that modulates the dynamics of the flexible [30,32] ε PL.

## 3. Discussion

Chronic HBV infection [2] imposes a heavy global burden that necessitates the discovery and design of novel antiviral drugs to complement existing IFN-α and NRTI treatments. One attractive therapeutic target is the ε–P binding interaction that initiates pgRNA–P packaging [13,25] and reverse transcription [26,27,28,29]. However, the lack of structural data on P prevents the structure-guided design of anti-HBV therapeutics. It is important to note that multiple retroviral-derived homology models of reverse transcriptase (RT) domains exist for HBV [59,60,61]. In addition, ab initio predicted models have been recently reported for the terminal protein (TP) domain [62] and for the entire P protein [63]. While these predicted structures [59,60,61,62,63] may provide valuable platforms for future drug discovery and design, experimentally derived structures are preferred. 

As an initial step in the direction of structure-informed anti-HBV therapeutic discovery, we carried out a VS against our recent solution NMR structure of FL ε [30] (Figure 1a,b) using an FDA-approved compound library, followed by in vitro binding assays, robust computational docking, and MD simulations. We designed our VS to rapidly identify potential lead compounds that had good predicted affinity, favorable drug-like properties, and that were likely to target a structural region of functional importance (e.g., the PL) (Figure 2). Our initial lead compounds were then experimentally validated with an in vitro dye-displacement-binding assay (Figure S2). Of our 12 VS-identified lead compounds (Figure 2e), only three of the anti-HCV compounds: Ledipasvir, Simeprevir, and Daclatasvir bound FL ε (Figure 3 and Figure S4). When tested against additional RNAs with similar structural features, only Daclatasvir selectively targeted FL ε (Figure 4 and Figure S5). As a preliminary means of mapping the specific binding site of Daclatasvir to FL ε, we employed our dye-displacement assay and NMR titrations using ε modular constructs (Figure 5a). These data suggest that Daclatasvir binding is localized to the LH and PL (Figure 5). To gain additional insight into the FL ε–Daclatasvir interaction, we carried out robust computational docking and MD simulations. This analysis revealed that Daclatasvir selectively targets the ε PL (Figure 6a) and remains stably bound throughout the MD run (Figure 6b), suggesting a valid docking pose prediction.

It is important to note that, by themselves, the individual binding and docking data are tentative. Firstly, in our dye-displacement assays, Daclatasvir and other antiviral compounds can bind the dye in the absence of RNA (Figure S3), leading to an internal fluorescence that results in a dampened binding response (i.e., minimal fluorescence attenuation) (Figures S4–S6; see Section 4). As such, fluorescence changes might not be additive, further complicating the interpretation of this assay. Secondly, while our NMR titrations (Figure 5c) agree with our dye-displacement (Figure 5b) data, most CSPs are small and experimentally restricted to non-PL nucleotides. Finally, our computational data have some notable caveats. To start, our VS strategy may have resulted in discarding real binders or overestimating the binding properties of some compounds. In addition, given that FL ε is highly dynamic [30,32], it would have been desirable to carry out our VS on a conformational ensemble of FL ε or on multiple NMR models. As such, all computational data by itself must be interpreted with caution. Nevertheless, our dye-displacement (Figure 5b), NMR titration (Figure 5c), docking (Figure 6a), and MD (Figure 6b–d) data all agree that Daclatasvir targets FL ε mainly at its PL (Figure S7). 

Importantly, a comparison of the MD data of unliganded and Daclatasvir-bound FL ε R3 suggests that Daclatasvir increases the flexibility and conformational variety of nucleotide U15 but rigidifies the motions of nucleotides U17-C19 (Figure 6c,d). Interestingly, both Raloxifene [30] and Daclatasvir modulate the dynamics of the flexible [30,32] ε PL, whose motions are likely critical for functioning [32]. As such, regulating ε dynamics may be an effective therapeutic strategy, which would benefit low affinity binders that are unlikely to outcompete P binding, given that RT domains have a low nM affinity for nucleic acids. Instead, dynamic-regulating small molecules can induce their effect by preventing ε from adopting the conformations needed to move from one functional state (i.e., P binding, pgRNA–P packaging, and reverse transcription) to the next. Indeed, considerations of RNA dynamics in small-molecule targeting have shown promising results in RNA-targeted drug discovery [39,40]. Therefore, even though Raloxifene has no anti-HBV effect and this information is not yet known for Daclatasvir, the approaches described herein provide a useful platform for the discovery of new compounds whose ability to alter ε dynamics may result in the inhibition of early stages of HBV replication.

## 4. Materials and Methods

### 4.1. Materials

All VS-identified compounds were purchased from ChemScene (Monmouth Junction, NJ, USA) (Elbasvir and Velpatasvir); APExBIO (Houston, TX, USA) (Daclatasvir, Natamycin, and Saquinavir); 1PlusChem (San Diego, CA, USA) (Ceftaroline fosamil, Folinic acid, and Simeprevir); and AKScientific (Union City, CA, USA) (Ivermectin, Ledipasvir, Minocycline, and Telithromycin) and used as-is. 

### 4.2. Virtual Screening

We carried out our VS with AutoDock Vina [54] in the PyRx open-source software package [55]. In brief, SDF files of our 1604-compound FDA-approved library were downloaded from the ZINC15 database [46,47,48] and loaded into PyRx with the Open Babel chemical toolbox [64]. The SDF files were then energy-minimized and appropriately protonated to generate the required PDBQT files. Once all ligands were prepared, FL ε R3 (PDB 6var) [30] was loaded and prepared as the receptor molecule. The docking grid was prepared in a manner to ensure an unbiased dock (i.e., the grid encompasses the entire receptor molecule), and therefore, dimensions of 64.9 × 57.0 × 39.3 were used. Finally, we enabled nine possible docking poses per ligand. The intention of our VS was to rapidly screen our compound library and rank-order our lead compounds by predicted affinity. We therefore carried out selection criteria on the basis of affinity, commercial availability and drug-like properties, and dock site (Figure 2a) to identify the lead compounds from our 1604-compound library. For our first selection step, we took the 122 compounds whose top-ranked docking pose had a predicted affinity higher than that of the already known [30] ε-targeting ligand Raloxifene (−9.5 kcal·mol^−1^) (Figure 2b). For our second selection step, we excluded all compounds that were not commercially available and/or had potential adverse effects and proceeded with 66 compounds (Figure 2c). This step required the manual curation of the compound library. We considered any anticancer drug or any compound with a mode of action that included the inhibition of fundamental cellular processes (e.g., DNA replication) as having a potential adverse effect. Given the functional importance of the PL [22,23,27,29,33,34] (Figure 1a) and our previous computational [30,31] (Figure 1c) and experimental [30] (Figure 1d) data, our final selection step chose compounds that targeted the ε PL. To increase our confidence that we proceeded with authentic ε PL-targeting compounds, we repeated our docking three additional times. Then, we classified confident PL docking on the basis of two criteria: (1) top-rank pose localized to the PL in >50% of the repeated runs and/or (2) >50% of all poses localized to the PL (Figure S1). In both instances, PL localization was loosely defined as having more than one contact within 5 Å of a PL nucleotide (i.e., C14–C19, including the adjacent A13, A20, U48, and U49). In total, our VS identified 12 initial lead compounds (Figure 2e). 

### 4.3. RNA Transcription

All RNA samples were prepared by T7 RNA polymerase (RNAP)-based in vitro transcription following well-established protocols [65]. In brief, transcriptions were carried out in 40 mM Tris-HCl (pH 8 at 37 °C), 1 mM spermidine, 0.01% Triton-X100, 80 mg/mL polyethylene glycol, 0.3 μM DNA templates (Integrated DNA Technology), 1 mM DTT, 2 U/µL thermostable inorganic pyrophosphatase (New England Biolabs, Ipswich, MA, USA), 5–15 mM ribonucleotide 5′-triphosphates (rNTPs), 5–15 mM MgCl_2_, and 0.1 mg/mL T7 RNAP. The reactions were first optimized for appropriate rNTP and MgCl_2_ concentrations at the small (50 µL) and medium (500 µL) scales before carrying out large-scale (5 mL) reactions. All transcriptions proceeded for 3 h at 37 °C. After transcription, the samples were extracted with acid phenol:chloroform, ethanol precipitated, purified by preparative denaturing polyacrylamide gel electrophoresis, and electroeluted. The samples were then dialyzed five times against UltraPure water and folded by heating for 2 min at 95 °C, snap-cooling on ice, and slowly equilibrating to room temperature. 

### 4.4. Dye-Displacement Assay

Our 12 VS-identified lead compounds were dissolved in either UltraPure H_2_O or DMSO (depending on their solubility) to make 0.1, 1.0, and 10.0 mM stock solutions. In all in vitro binding assays, a fixed concentration of FL ε (0.5 µM) and SYBRG II (4×) (Millipore Sigma) was used. Then, 5 µL of compound (in 100% DMSO or H_2_O) and 95 µL of RNA-dye complex in the assay buffer (5 mM sodium cacodylate pH 6.5, 50 mM KCl, 1 mM MgCl_2_, 0.1 mM EDTA, and 0.01% Triton-X100) were added to black Costar 96-well plates; incubated at room temperature for 30 min; and the fluorescence intensity values were measured (485 ± 5 nm excitation, 525 ± 5 nm emission) using a SpectraMax M5 (Molecular Devices) plate reader equipped with SoftMax Pro analysis software. The initial experiments were performed with 500 µM of each compound to determine which ligands yielded fluorescence attenuation. Follow-up experiments to quantify binding were then carried out by titrating increasing concentrations of each compound against various RNAs. In such experiments, the EC_50_ values were determined by normalizing the fluorescence intensity of each well to an average value for the fluorescence intensity of the RNA–dye complex by the following relation:

(1) $${Y=F}_{\min}+\frac{\left(F_{\max}-{\;F}_{\min}\right)}{\left({(1+10}^{{\log(\mathrm{EC}}_{50}-\;X)\cdot \mathrm{HillSlope}}\right)}$$

where F_max_ and F_min_ are the highest and lowest fluorescence readings, HillSlope is the steepness (i.e., responsiveness) of the curve, X is the logarithm of the ligand concentration, and Y is the normalized fluorescence [30]. The reported EC_50_ values are the average ± standard error from the nonlinear regression fitting of data from triplicate measurements to Equation 1 using MATLAB (version 2019a).

It is important to note that a subset of the large and highly aromatic compounds tested (i.e., antivirals: Daclatasvir, Elbasvir, Ledipasvir, Saquinavir, Simeprevir, and Velpatasvir) showed RNA-independent binding to the dye, leading to an increase in fluorescence (Figure S3). We therefore included control wells on each plate that only contained the ligand and dye without RNA (Figure S3), which was incorporated into their fluorescence normalization. For some of these dye-binding compounds, the resulting binding curves showed minimal fluorescence attenuation (e.g., ~15%) and a dampened response (e.g., Daclatasvir in Figures S4–S6). The same was not true for other dye-binding compounds, which showed a more typical binding curve (e.g., Simeprevir in Figures S4 and S5). Even when a dampened response was observed, binding curves that show binding (e.g., Daclatasvir with FL ε and PL ε in Figures S4 and S6) are markedly different than those from non-binding events (e.g., Daclatasvir with AL ε in Figure S6). Nevertheless, the analysis of these data is not straightforward, and the derived EC_50_ values likely do not reflect accurate binding affinities and should therefore be interpreted with caution. 

### 4.5. NMR Titrations

All ε NMR samples were prepared by in vitro transcription (as described in Section 4.3) and were dialyzed into the NMR buffer (10 mM Na_3_PO_4_, pH 6.7, and 0.1 mM EDTA). NMR titration experiments were performed on unlabeled FL ε (Figure 1a) and modular constructs PL ε and AL ε (Figure 5a). Daclatasvir was dissolved in DMSO-d_6_ to make a 10 mM stock solution. Daclatasvir (100 µM) was screened by titrating against ε samples (50 µM), and therefore, the final NMR samples contained 1% DMSO-d_6_. ^1^H NMR experiments were used to monitor CSPs of imino protons (i.e., guanosine-H1 and uridine-H3). All NMR data were collected on an Avance III Bruker Ultrashield 600 MHz spectrometer equipped with a triple-resonance cryogenic probe. Spectra were collected at 25 °C with a recycle delay of 1.5 s and analyzed using TopSpin 4.0. 

### 4.6. Computational Docking

Initial mapping of probable ligand cavities in FL ε was carried out with the machine learning tool RNACavityMiner [31] using FL ε R3 (PDB 6var) [30] as the target (Figure 1c). Later on, rDock [57] was used to predict the Daclatasvir docking pose to FL ε R3 [30]. This program offers a dedicated intermolecular scoring function (e.g., van der Waals, polar, and desolvation components) that has been validated against RNA targets [57]. First, rbcavity generates the docking cavity for the receptor (i.e., docking surface interface). Then, rbdock docks the ligand. rDock-predicted ligand pose predictions are based on sampling of the exocyclic dihedral angles that yield the best docking scores when fit to a rigid target (i.e., receptor [30]). The program employs a genetic algorithm-based stochastic search algorithm and therefore must be run multiple times. rbdock was run 10 times to generate the top-ranked docking poses. As before, our docking grid was prepared to ensure an unbiased dock, and therefore, a search radius of 0.0 Å was used (i.e., the search was not restricted). The receptor input was converted to a MOL2 format, while the ligand conformations were converted to a SDF format. The Daclatasvir docking pose predictions generated by rDock were then rescored by RNAPosers [58], a machine learning pose classifier of RNA–ligand complexes. Given a receptor file (e.g., FL ε R3, PDB 6var [30]) and a file containing ligand poses (e.g., those generated from rDock), RNAPosers returns the relative classification scores to predict the pose that is most near-native [58]. The top-scored Daclatasvir docking pose derived from RNAPosers [58] selectively targets the ε PL (Figure 6a). Moreover, all 10 predicted poses dock to the ε PL with a strong overall agreement (Figure S9), suggestive of an accurate prediction. rDock, RNAPosers, and RNACavityMiner were all accessed through the SMALTR Gateway at https://smaltr.org/ (accessed on 4 November 2021). 

### 4.7. Molecular Dynamic Simulations

The Amber20 software package [66] was used to perform MD simulations with the ff99LJbb [67] force field (source file leaprc.RNA.LJbb), which combines the OL3 [68] parameter set, the Steinbrecher and Case phosphate oxygen van der Waals radii [69], and the OPC water model [70,71]. The Amber antechamber package was used to generate a standard MOL2 file for Daclatasvir with 3D coordinates and atom types matched to the general force field GAFF. Antechamber also generated the ligand library PREP file, while the Amber utility parmchk2 was used to generate a FCRMOD file that contains any force field parameters not listed in GAFF. The GAFF, PREP, FRCMOD, and ligand (Daclatasvir) and receptor (FL ε R3, PDB 6var [30]) PDB files were input into the Amber LEaP module, which combines them with OPC waters, Joung-Cheatham [72] monovalent ions (Na^+^/Cl^−^), and the RNA-specific force field parameters mentioned above to generate the topology and coordinate files. 

Explicit solvent molecular particle mesh Ewald dynamics simulations were utilized [73]. FL ε R3 and the FL ε R3–Daclatasvir complex were placed in a cuboid solvent box with OPC waters, and the minimum distance between the solute and solvent box boundary was set at 12 Å. The net solute charge was neutralized with Na^+^ ions, and additional Na^+^/Cl^−^ ion pairs were added to simulate a 0.15 M salt concentration for the entire system. Simulations were run with 2.0 fs time steps, employing the SHAKE algorithm to constrain all hydrogen bonds. The Berendsen thermostat [74] and algorithm were used to maintain the simulation temperature at 300 K and to maintain the pressure at 1.0 Pa in the NPT simulations used in all phases of the MD. A cutoff of 9 Å for the nonbonded interactions was used, and explicit solvent periodic boundary conditions were employed. 

A 12-step equilibration protocol was used in all simulations that started with energy minimization of the solvent (while FL ε R3 and FL ε R3–Daclatasvir were restrained), followed by multiple short phases of heating to 300 K, dynamics at 300 K, and energy minimizations with gradually decreasing harmonic restraints applied to the solute. The last phase of the equilibration protocol was an unrestrained heating to 300 K, ramped up over 0.2 ns and kept at the steady target temperature for a total time of 2.0 ns. Unrestrained MD simulations were performed for 500 ns on FL ε R3 and FL ε R3–Daclatasvir, and the Amber CPPTRAJ [75] module was used for analysis. The 500 ns MD trajectories were sampled every 0.1 ns to yield 5000 data points. The magnitude of the RNA structural motions (distortions) in MD was such that aligning the MD-sampled conformers to the starting NMR reference model would yield meaningless root mean square fluctuation (RMSF) values for individual nucleotides. Therefore, local fluctuations were monitored by calculating all atom RMSDs within a 3-nt sliding window (ranging from G1-U3 to A59-C61). These RMSF-like data (excluding the first 25 ns of equilibration) were then plotted (from 26-500 ns) for the positions of central nucleotides (i.e., from G2-C60) (Figure 6c,d).

## 5. Conclusions

In summary, we employed a structure-informed VS, followed by in vitro binding assays, to identify ε-targeting ligands from a 1604 FDA-approved compound library that may serve as novel anti-HBV therapeutics. This approach revealed that the anti-HCV drug Daclatasvir is a selective ε-targeting ligand. Additional computational docking and MD simulations demonstrated that Daclatasvir targets ε at its flexible [30,32] PL and modulates its dynamics. Taken together, our work supports the notion that targeting ε dynamics may be an effective anti-HBV therapeutic strategy. However, confirmation of this hypothesis requires testing whether Daclatasvir can prevent early stages of HBV replication in vitro and in vivo.

## Data Availability

Data are contained within the article or the Supplementary Materials.

## Supplementary Materials

The following supporting information can be downloaded at: https://www.mdpi.com/article/10.3390/molecules28041803/s1: Figure S1: Full-length (FL) ε R3 priming loop (PL) docking validation. Figure S2: Schematic of our dye-displacement assay. Figure S3: Dye-displacement data for antiviral compounds that bind SYBR Green II. Figure S4: Binding (and non-binding) of all 12 VS-identified lead compounds. Figure S5: Selectively test of binding to FL ε. Figure S6: Binding of Daclatasvir to ε modular constructs. Figure S7: Summary of in vitro binding data and computational docking of Daclatasvir and FL ε. Figure S8. Initial modeling of the FL ε R3–Daclatasvir interaction. Figure S9: Daclatasvir selectively docks to the FL ε R3 PL.

## Abbreviations

HBV: hepatitis B virus; HCC: hepatocellular carcinoma; FDA: Food and Drug Administration; IFN-α: interferon-α, NRTI(s): nucleos(t)ide reverse transcriptase inhibitor(s); P: HBV polymerase protein; nt: nucleotide; pgRNA: pre-genomic RNA; NMR: nuclear magnetic resonance; FL: full-length; PL: priming loop; SERM(s): selective estrogen receptor modulator(s); VS: virtual screen; HCV: hepatitis C virus; MD: molecular dynamics; RDCs: residual dipolar couplings; NOEs: Nuclear Overhauser effect; ADMET: absorption, distribution, metabolism, excretion, and toxicity; HIV: human immunodeficiency virus; EC_50_: half maximal effective concentration; A-site: 27-nt RNA from the decoding center of Escherichia coli ribosomal RNA; TAR-2: 30-nt RNA from the transactive response element from HIV-1; D5-PL: 34-nt RNA from the self-splicing group II intron catalytic effector domain 5 from Pylaiella littoralis; LH: lower helix; AL: apical loop; UH: upper helix; PTL: pseudo-tri-loop; CSP(s): chemical shift perturbation(s); RMSD: root-mean-square deviation; RT: reverse transcriptase; rNTPs: ribonucleotide 5′-triphosphate(s); RMSF: root-mean-square fluctuation.
