# Supplementary material for: Virtual Screening of Hepatitis B Virus Pre-Genomic RNA as a Novel Therapeutic Target
Source: Molecules. 2023 Feb 14;28(4):1803. doi: 10.3390/molecules28041803 (PMC9963113; doi:10.3390/molecules28041803)
Supplement: Supplementary file 1 [file molecules-28-01803-s001.zip › molecules-2183589_supplementary.pdf]

Supplementary Material

to

Virtual screening of hepatitis B virus pre-genomic RNA as a novel therapeutic target

Lukasz T. Olenginski[1], Wojciech K. Kasprzak[2], Solomon Attionu[1], Bruce A. Shapiro[3], and Theodore K. Dayie[1]*

1 - Department of Chemistry and Biochemistry, University of Maryland, College Park, MD 20742, United States.
2 - Basic Science Program, Frederick National Laboratory for Cancer Research, Frederick, MD 21702, United States.
3 - RNA Biology Laboratory, National Cancer Institute, Frederick, MD 21702, United States

***Corresponding author email: dayie@umd.edu.

This file includes:

Supplementary Figures S1 to S9

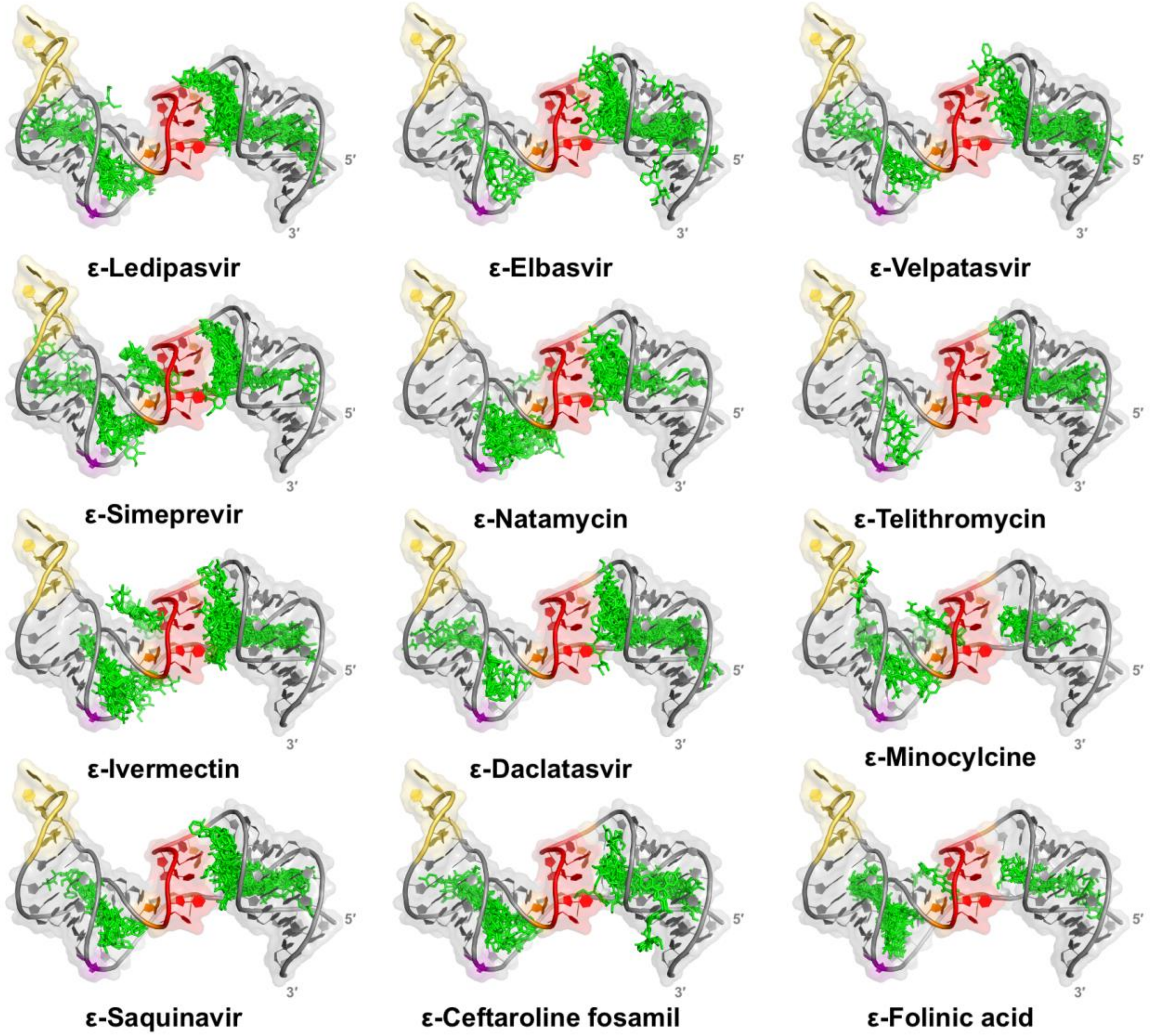

Supplementary Figure S1. Full-length (FL) ε R3 priming loop (PL) docking validation. FL ε R3-lead compound complexes of all ligand poses in all docking runs with the FL ε R3 (PDB 6var) [1] target. ε structural regions are abbreviated and colored as in Figure 1a and all compounds are shown in green sticks. Each of the 12 compounds passed the final selection step of our virtual screen (VS), which determined confident PL docking on the basis of two criteria: (1) top-rank pose localized to the PL in >50% of repeated runs and/or (2) >50% of all poses localize to the PL. In both instances, PL localization is loosely defined as having more than one contact within 5 Å of a PL nucleotide (i.e., C14-C19, including the adjacent A13, A20, U48 and U49). As is more evident for certain compounds, our loose criteria result in some instances where PL docking is not immediately visible. Nevertheless, given that we planned to validate the results of our VS with *in vitro* binding assays, we were satisfied with our final selection step.

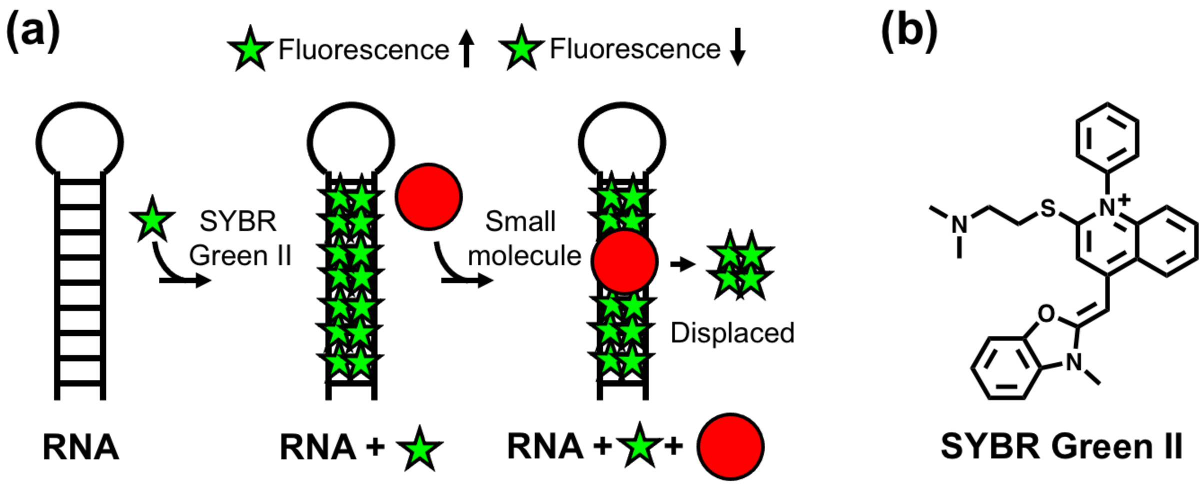

Supplementary Figure S2. Schematic of our dye-displacement assay. (a) Representation of the *in vitro* dye-displacement experiment used to probe compound competitive binding to RNAs. Saturation of RNA with the intercalator SYBR Green II causes an increase in dye fluorescence. Small molecule binding to RNA can therefore displace the dye, leading to fluorescence attenuation. (b) Structure of SYBR Green II.

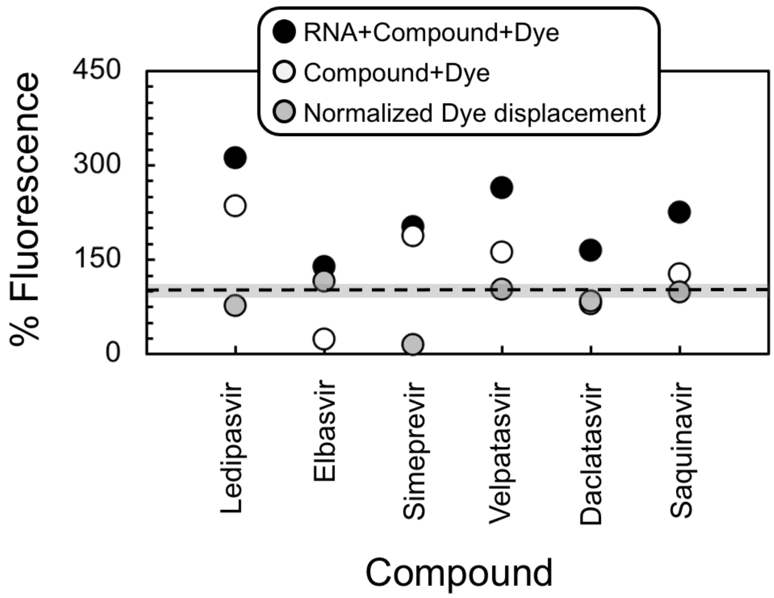

Supplementary Figure S3. Dye-displacement data for antiviral compounds that bind SYBR Green II. Data are shown for conditions with RNA, dye, and compound (black points) as well as for a no-RNA control (i.e., just compound and dye) (white points). % Fluorescence >100% for the former condition suggests that that these compounds either enhance the interaction between FL ε and the dye or bind the dye themselves. To establish fluorescence decreases that are specifically attributable to compounds that bind FL ε, the no-RNA fluorescence signals were subtracted from the conditions with RNA. These normalized data points are shown in gray. % Fluorescence of the normalized data of ≥100% (shown by the dashed line) indicates that the compound does not bind FL ε whereas % Fluorescence <100% indicates that the compound do bind FL ε and displaces SYBR Green II. To avoid false positives, binding compounds were selected if they lead to >10% fluorescence attenuation (show by the lower shaded region). Ledipasvir, Simeprevir, and Daclatasvir all show displacement of SYBR Green II indicative of FL ε binding.

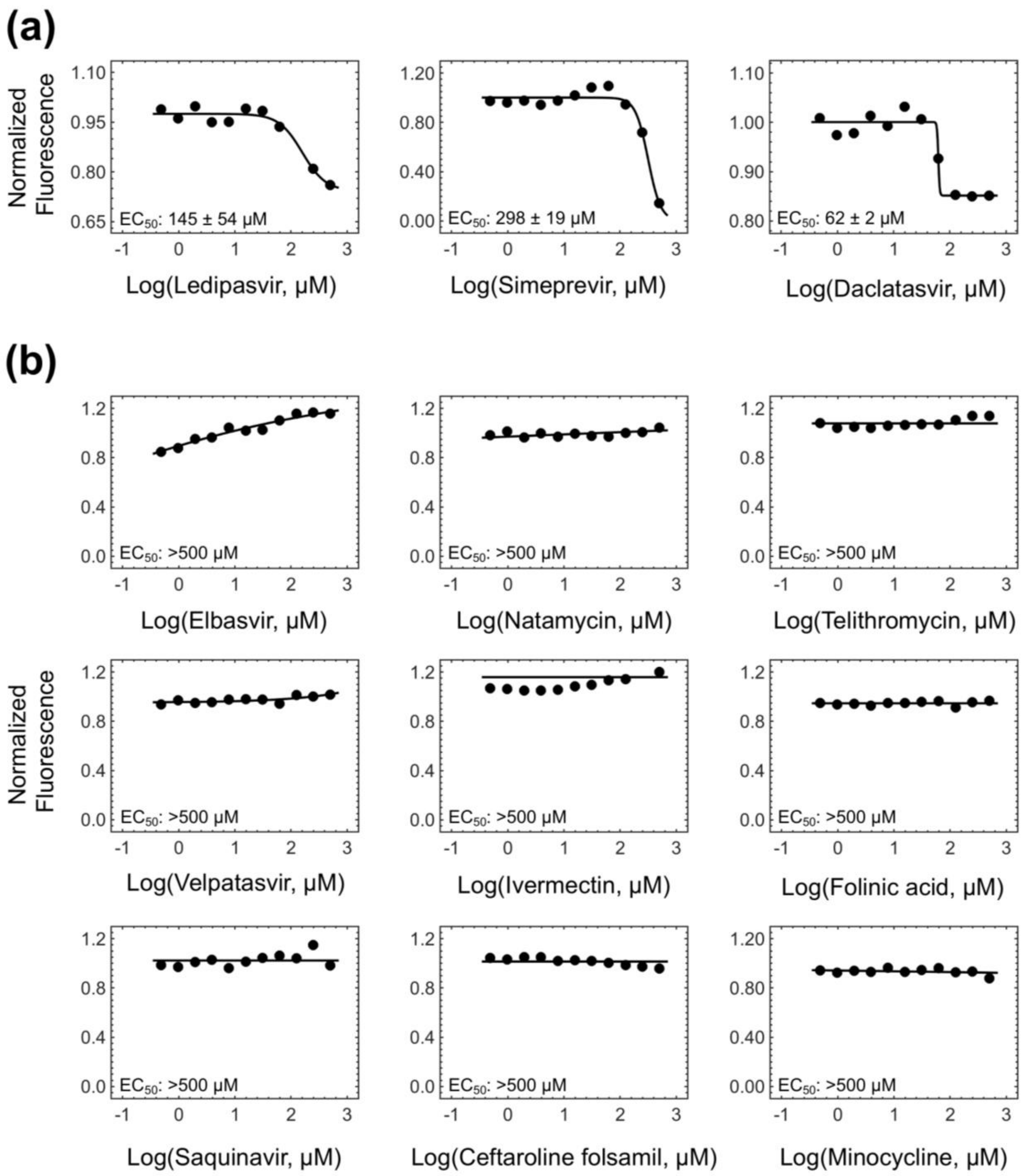

Supplementary Figure S4. Binding (and non-binding) of all 12 VS-identified lead compounds. (a) Representative binding curves from triplicate dye-displacement experiments for (a) binding and (b) non-binding VS-identified lead compounds and FL ε. Compounds in (a) show evidence of binding. Half maximal effective concentration (EC_{50}) values are reported as the average ± standard error (SE) from fitting data to Equation 1 (see Materials and Methods). Different scales are used on the y-axes because these large and highly aromatic compounds bind the dye, leading to dampened responses that vary the attenuation in dye fluorescence (Figure S3, see Materials and Methods). Compounds in (b) do not show evidence of binding at the concentrations used (i.e., EC_{50} >500 μM), as determined by data fitting to Equation 1.

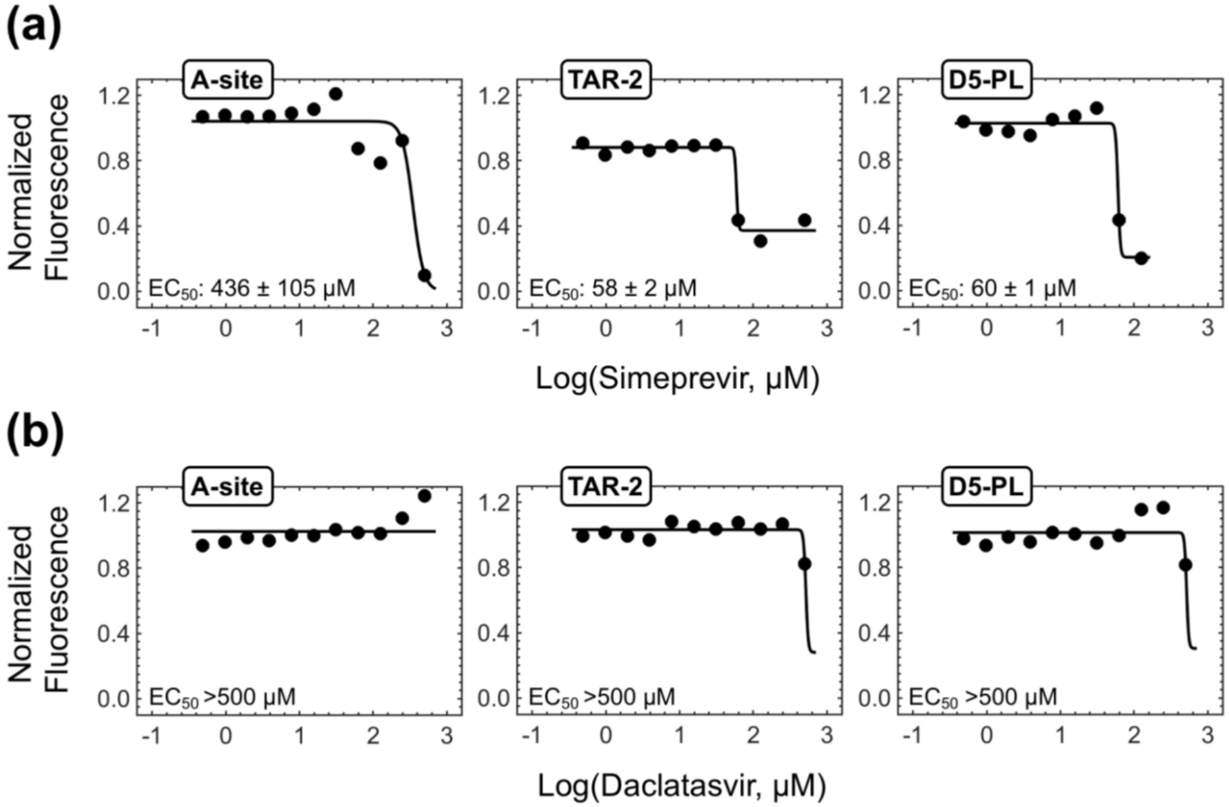

Supplementary Figure S5. Selectively test of binding to FL ε. Representative binding curves from triplicate dye-displacement experiments for (a) Simeprevir and (b) Daclatasvir and A-site, TAR-2, and D5-PL RNAs (Figure 4a). EC_{50} values are reported as the average ± SE from fitting data to Equation 1. Only Daclatasvir shows evidence of selective binding to FL ε.

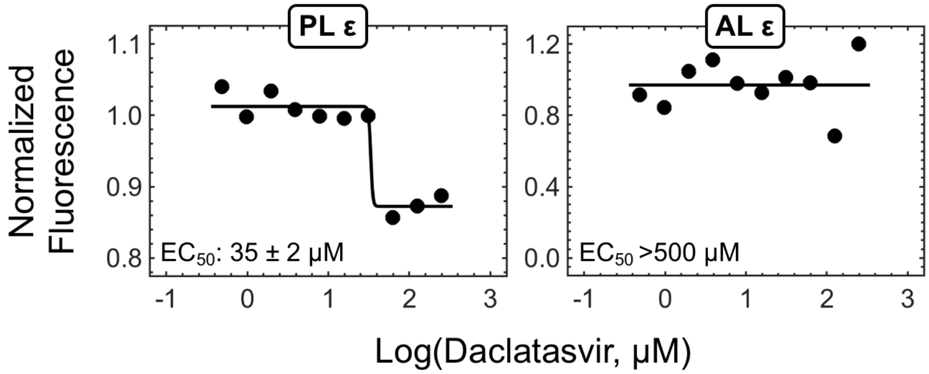

Supplementary Figure S6. Binding of Daclatasvir to ε modular constructs. Representative binding curves from triplicate dye-displacement experiments for Daclatasvir and PL ε and AL ε RNAs (Figure 5a). EC_{50} values are reported as the average ± SE from fitting data to Equation 1. Daclatasvir only shows evidence of binding FL ε and PL ε , suggesting that its binding is localized to regions shared between the two constructs: the LH and PL.

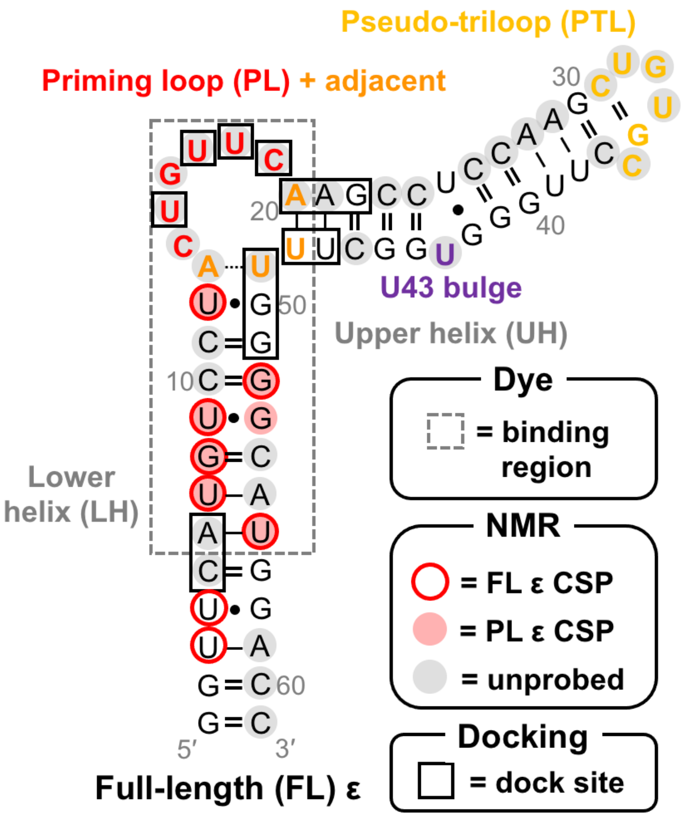

Supplementary Figure S7. Summary of *in vitro* binding data and computational docking of Daclatasvir and FL ε. Representative data from dye-displacement (Figures 3 and 5b), NMR titration (Figure 5c) and computational docking (Figure 6a) analysis are shown mapped onto the secondary structure of FL ε. Structural regions are abbreviated and colored as in Figure 1a. Taken together, our data all agree that Daclatasvir targets FL ε mainly at its PL.

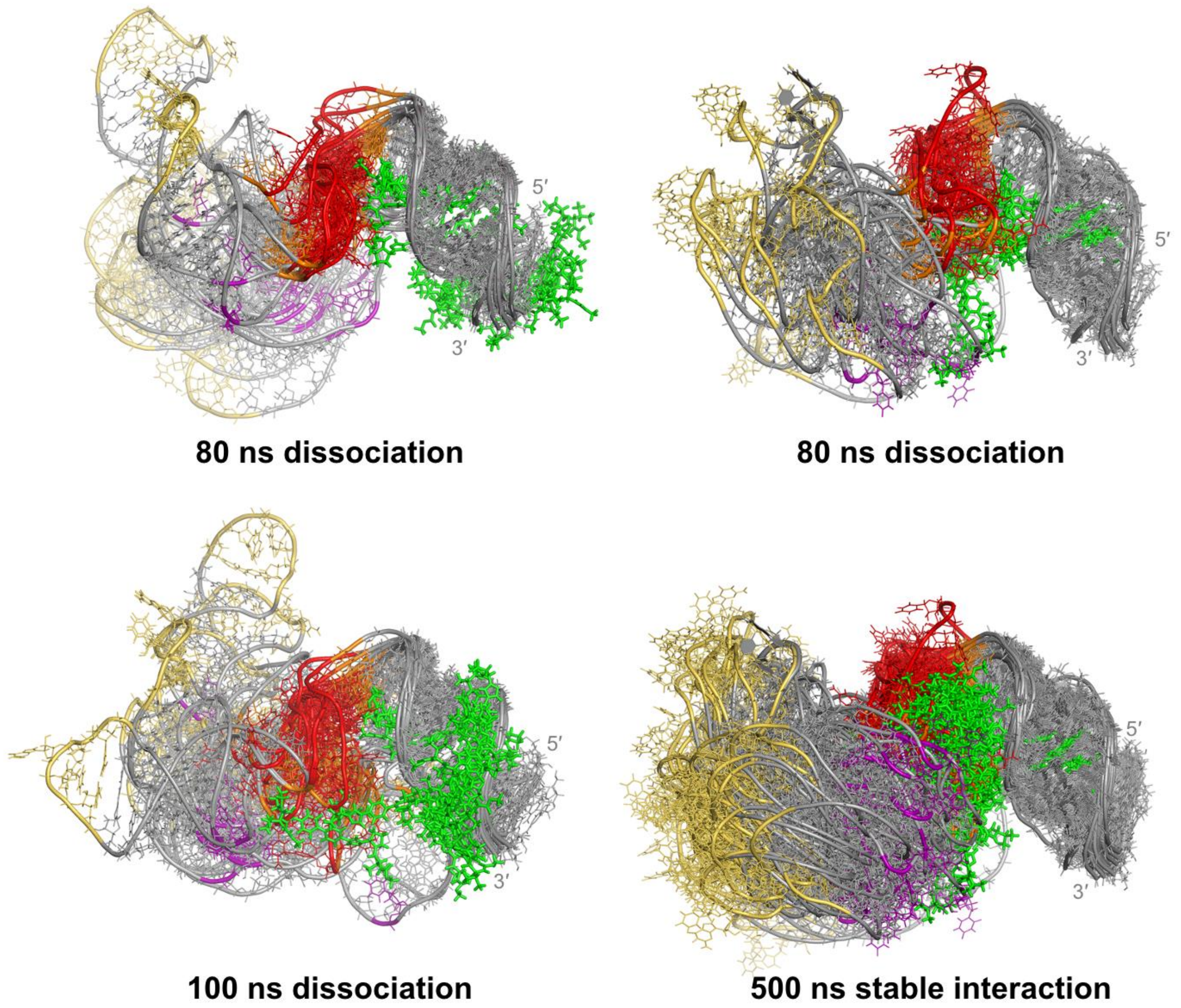

Supplementary Figure S8. Initial modeling of the FL ε R3-Daclatasvir interaction. Molecular dynamic (MD) simulations of the top-ranked Daclatasvir pose derived from AutoDock Vina. Structural overlay of PDB snapshots taken every 10 ns of FL ε R3-Daclatasvir MD trajectories of varied lengths. ε structural regions are abbreviated and colored as in Figure 1a and Daclatasvir is shown in green sticks. Structural ensembles are shown with backbone phosphorus atom alignments using LH nucleotides (i.e., G1-U12 and G50-C61). Three out of four simulations yielded quick (i.e., 80-100 ns) Daclatasvir dissociations whereas one resulted in a stable complex for the entire 500 ns MD run.

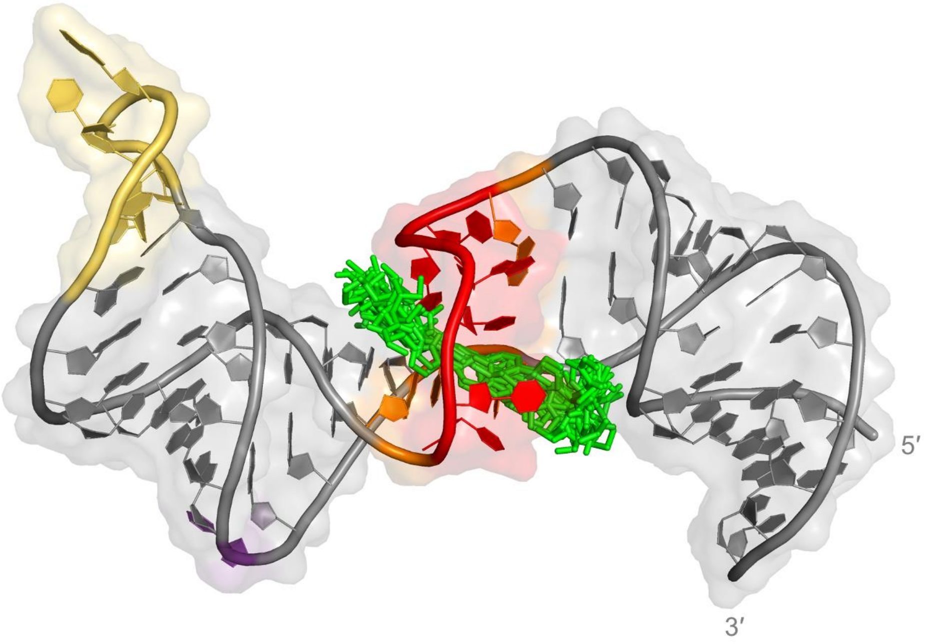

Supplementary Figure S9. Daclatasvir selectively docks to the FL ε R3 PL. All 10 rDock [2] predicted Daclatasvir docking poses to FL ε R3 (PDB 6var) [1] are shown in green sticks. ε structural regions are abbreviated and colored as in Figure 1a. All predicted poses dock to the ε PL with strong overall agreement, suggestive of an accurate prediction.

References

1. LeBlanc, R.M.; Kasprzak, W.K.; Longhini, A.P.; Olenginski, L.T.; Abulwerdi, F.; Ginocchio, S.; Shields, B.; Nyman, J.; Svirydava, M.; Del Vecchio, C.; et al. Structural insights of the conserved "priming loop" of hepatitis B virus pre-genomic RNA. *J. Biomol. Struct. Dyn.* **2022**, *40,* 9761–9773.
2. Ruiz-Carmona, S.; Alvarez-Garcia, D.; Foloppe, N.; Garmendia-Doval, A.B.; Juhos, S.; Schmidtke, P.; Barril, X.; Hubbard, R.E.; Morley, S.D. rDock: a fast, versatile and open source program for docking ligands to proteins and nucleic acids. *PLoS Comput. Biol.* **2014**, *10*, e1003571.
